# New therapies

**DOI:** 10.1186/ar3938

**Published:** 2012-09-27

**Authors:** David Wofsy

**Affiliations:** 1 University of California, San Francisco, CA, USA

## 

The past decade has brought unprecedented progress in the refinement of conventional therapies for systemic lupus erythematosus (SLE) and the development of biologic therapies for SLE. Extensive recent evidence has expanded our understanding of the potential benefits of antimalarial therapy and has demonstrated improved approaches to the use of cyclophosphamide. Concurrently, a strong foundation of evidence has been generated to support the use of mycophenolate mofetil, especially in lupus nephritis.

Against this background, there has been mounting excitement surrounding the promise of biologic therapies. Belimumab demonstrated efficacy in two phase III trials involving patients with diverse, nonrenal non-CNS, manifestations of SLE. The success of these trials has drawn attention to a novel primary endpoint, the SLE Responder Index (SRI). In this regard, it bears emphasizing that the trials were positive because the drug had a demonstrable effect, not because of the novelty of the endpoint. Indeed, the SLEDAI component of the outcome measure distinguished the treatment groups from the control groups with virtually the same statistical certainty as the SRI. Therefore, it remains to be determined which, if either, of these outcome measures might perform best in future lupus trials. At present, numerous follow-up trials are underway to assess which patient subpopulations and which disease manifestations are responsive to belimumab.

At the same time that belimumab was being tested in patients with lupus manifestations other than nephritis, abatacept was being tested in patients with lupus nephritis. A large international trial failed to achieve its primary endpoint. However, *post-hoc *analyses have raised questions about that result. Specifically, when the data from the abatacept trial were subjected to the outcome measures from other major lupus nephritis trials (LUNAR and ALMS), the complete response (CR) rates among subjects treated with abatacept were substantially higher than the CR rates in the control group [1]. Subsequent analyses of the data from this trial examined the discriminatory capability of other possible outcome measures and demonstrated that, at least in this dataset, the CR rate at 12 months discriminated treatment from control groups more effectively than other common outcome measures (including partial response (PR), overall response (CR+PR), treatment failure rate, or response rates at 6 months rather than 12 months). Studies currently in progress should help to clarify whether abatacept is effective in the treatment of lupus nephritis.
